# Development of Amperometric Hydrogen Peroxide Sensor Based on Horseradish Peroxidase-Immobilized Poly(Thiophene-*co*-EpoxyThiophene)

**DOI:** 10.3390/s8074110

**Published:** 2008-07-09

**Authors:** Hwa-Jung Kim, Ming-Hua Piao, Seong-Ho Choi, Chang-Ho Shin, Young-Taeg Lee

**Affiliations:** 1 Department of Chemistry, BK 21 NanoBiosensor Research Team, Hannam University, Daejeon 305-811, Republic of Korea; 2 KT&G Central Research Institute, 305-805, Republic of Korea

**Keywords:** Poly(Th-*co*-EpoxyTh) electrode, Horseradish peroxidase, Cyclic voltammetry, H_2_O_2_

## Abstract

A modified electrode for hydrogen peroxide (H_2_O_2_) sensing was prepared via thiophene (Th) with epoxy group. Thiophene (EpoxyTh) with epoxy group was synthesized by reaction of 3-bromothiophene and glycidyl methacrylate (GMA) in acetonitrile according to Heck Reaction. The electrocopolymerization of Th and EpoxyTh was performed on the surface of indium tin oxide (ITO) electrode by cycling the potential between -1.0 and +2.5 V in mixture of thiophene (Th) and EpoxyTh. Poly(Th-*co*-EpoxyTh) grown onto the ITO electrode was successfully confirmed by SEM, AFM, and water contact angle analysis, respectively. Finally, the HRP was immobilized on the surface of poly(Th-*co*-EpoxyTh) electrode by covalent binding. The amperometric response of the HRP-immobilized poly(Th-*co*-EpoxyTh) electrode for H_2_O_2_ was examined by cyclic voltammetry (CV). The HRP-immobilized poly(Th-*co*-EpoxyTh) electrode showed linearity from 0.1 to 30 mM H_2_O_2_, good reproducibility, and long life time.

## 1. Introduction

Biosensors based on small devices have been used in clinical analysis, environmental monitoring, and bioprocess control [1]. Enzyme immobilized electrodes have been used as biosensors [2-4]. Thus, there has been considerable attention in the immobilization of redox enzymes on the surface of electrode for electrochemical biosensors. These enzyme-based amperometric biosensors show high specificity and sensitivity in analysis of hydrogen peroxide (H_2_O_2_) [2-4]. H_2_O_2_ is not only a by-product of diverse substances under the catalysis of their highly selective oxidases, but also an essential mediator in food, pharmaceutical, clinical, industrial, and environmental analyses [5,6]. Accordingly, the determination of H_2_O_2_ is very important in the field of industrial, biological, food, pharmaceutical, clinical, and environmental analyses.

Horseradish peroxidase (HRP) is the most widely studied member of the peroxidase family in enzyme based biosensors. HRP contains heme as the prosthetic group, which is also the protein active site along with the heme-iron: Fe (III), that can catalyze the H_2_O_2_ dependent (one-electron) oxidation [7]. The HRP readily combines with H_2_O_2_ and the resultant (HRP-H_2_O_2_) complex can oxidize a wide variety of hydrogen donors [8].

The electro-polymerization of the conducting monomers has been studied extensively for the construction of biosensors, because the prepared conducting polymers have the excellent electrocatalytic properties and rapid electron transfer ability. Also, the conducting polymers permit the transfer of charges to produce electrochemical signals between the electrode and the incorporated biomolecules [9-12]. For biosensor applications, the conducting polymers are often functionalized with carboxyl, amino, aldehyde or succinimidyl carbonate groups, or conjugated directly with various electronic mediators or bio-recognizable molecules to facilitate immobilization.

In our previous paper, we synthesized the polythiophene with carboxylic acid groups in order to immobilize HRP via the copolymerization of thiophene and 3-thiopheneacetic acid in acetonitrile [13]. However, the amounts of HRP on the surface of polythiophene electrode with carboxylic acid groups are very low. We used the Au nanoparticle as spacer in order to increase the immobilization amounts [14], but the lifetime of HRP electrode for H_2_O_2_ sensing is short, because the HRP is degraded on the surface of polymer electrode.

For the efficient immobilization of enzymes to increase operational stability, covalent attachments to activated supports have been frequently used for enzyme immobilization. To induce covalent linkage with enzymes, epoxy-activated supports are presumably the most accessible ones to perform very easy immobilization. In this sense, supports containing epoxy groups seem to be useful to generate very intense multipoint covalent attachment with different nucleophiles placed on the surface of enzyme molecules. On the other hand, the thiophene has an environmental stability in doped and undoped states together with the structural adaptability. However, there are only few reports about poly(Th)-based polymer biosensor [13,14]. Moreover, there was no report for using materials of the thiophen with epxoy group.

In this paper, we describe the utility of film of poly(Th-*co*-EpoxyTh) immobilized with HRP through covalent bonding as sensor electrode. Poly(Th-*co*-EpoxyTh) grown onto the ITO electrode was successfully confirmed by SEM, AFM, and water contact angle analysis, respectively. The amperometric response of the HRP-immobilized poly(Th-*co*-EpoxyTh) electrode for H_2_O_2_ was examined by CV.

## 2. Experimental Section

### 2.1. Chemicals

Thiophene (Th), tetrabutylammoniumhexafluorophosphate (TBAF_6_P), and horseradish peroxidase (HRP, E.C. 1.11.1.7, Type VI, RZ > 3.0, >250 U/mg) were purchased from the Aldrich–Sigma chemical Co (USA). All other chemicals were including 30 % hydrogen peroxide solution, and buffer media purchased from OCI (Seoul, Korea). A fresh solution of H_2_O_2_ was prepared daily and used. Solutions for the experiments were prepared with water purified in a Milli-Q puls water purification system (Millipore Co. Ltd., the final resistance of water was 18.2 MΩcm^−1^) and degassed prior to each measurement.

### 2.2. Synthesis of EpoxyTh

Glycidyl methacrylated thiophene (EpoxyTh) was synthesized by Heck Reaction. In detailed synthesis condition was as follows. The EpoxyTh was synthesized by reaction of 3-bromo thiophene (4.0 mmol) and glycidyl methacrylate (5.0 mmol) in DMF (30 mL) with Et_3_N (6.0mmol) in the presence of Pd/C catalyst (0.012mmol) at 140 °C for 3 hrs. The conversion yield was ∼ 80.1% determined by HPLC with ODS column using MeOH as mobile phase. Spectrascopic data of EpoxyTh: ^1^H NMR (CDCl_3_, TMS): δ 7.7-7.3 (s and d, 3H, Th), 7.2 (s, 1H, Th-CH=), 4.3 (d, 2H, O-CH_2−_), 3.3 (t, 1H, epoxy), 3.2 (d, 2H, epoxy), 1.9 (s, 3H, -CH_3_); FT-IR (KBr): 1700 (CO), 1633 (C=C), 1263 (C-O) cm^−1^; GC-MASS: 224. 1.

### 2.3. Preparation of HRP immobilized electrode

**Scheme 1.**
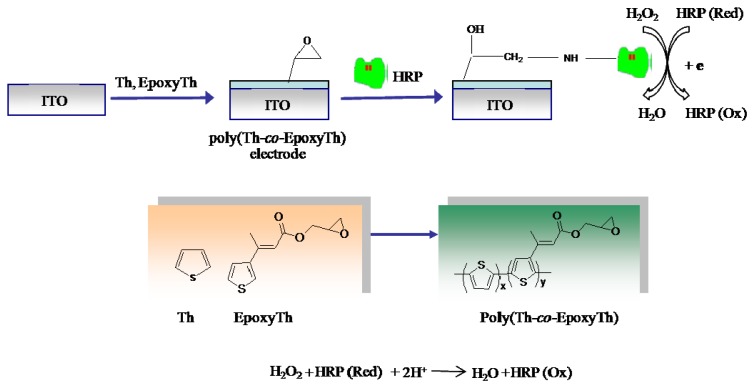
Preparation of H_2_O_2_ sensor by HRP immobilized poly(Th-*co*-EpoxyTh) electrode.

Scheme 1 describes the procedure for the HRP immobilized poly(thiophene-*co*-epoxythiophene) (poly(Th-*co*-EpoxyTh)) electrode. poly(Th-*co*-EpoxyTh) was coated over ITO electrode by electro-copolymerization of a mixture of thiophene (Th) and EpoxyTh by cycling the potential between -1.0 V to +2.5 V in acetonitrile containing TBAF_6_P as the background electrolyte. All experiments was carried out with a conventional three-electrode system having ITO (working area 0.7 × 1.1 cm^2^, 10Ω resistance, Korea) as the working electrode, a platinum wire as the auxiliary electrode, and an Ag/Ag^+^ (containing 0.1 M acetonitrile) electrode as the reference electrode. Electro-oxidative polymerization and copolymerization were carried out in acetonitrile containing 0.05 M of TBAF_6_P at a scan rate of 50 mV s^−1^. Then HRP was covalently immobilized onto the poly(Th-*co*-EpoxyTh) electrode by dipping the electrode in a 0.6 mg/ml HRP solution after acid-induced epoxide ring opening in poly(Th-*co*-EpoxyTh) [15] as shown in Scheme 1, and the electrode was stored at 4 °C prior to use.

### 2.4. Instrumentation

The surface morphology of the samples was determined by using field emission-scanning electron microscopy (FE-SEM, JSM-7000F, JEOL Ltd., Japan) and atomic force microscopy (AFM, PICO Station, Germany). Cyclic voltammetry was performed at 25 °C using a Potentiostat/Galvanostat model 283 (Ametek PAR, U.S.A). Fourier transform infrared (FTIR) spectra were recorded in the range 400-4000 cm^−1^ with a 4 cm^−1^ resolution from KBr pellets on a Perkin-Elmer Spectrum 1000 system (Perkin-Elmer life and analytical sciences, USA). Also, contact angle measurements were performed using a Phoenix 300 goniometer (Surface Electro Optics Co., Ltd., Korea).

## 3. Results and Discussion

**Figure 1.**
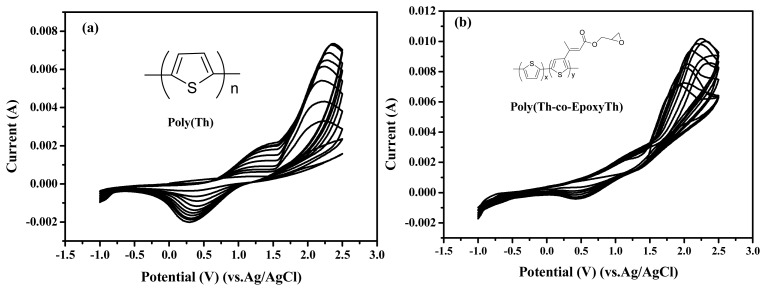
Cyclic voltammograms of polymers during electropolymerization in acetonitrile sol'n. poly(Th) (a), and poly(Th-*co*-EpoxyTh) (b).

Figure 1 displays the cyclic voltammograms recorded during the electrochemical polymerization of electrochemical polymerization of thiophene (50 mM) on ITO electrode (a), and electrochemical copolymerization of comonomers with thiophene and EpoxyTh on ITO electrode (b) in acetonitrile electrolyte containing 0.05M TBAF_6_P. The molar ratio of Th:EpoxyTh was to be 1:0.1. In Fig. 1(a), the starting oxidation peak of thiophene was appeared at +1.6 V, and the doping/dedoping processes of poly(Th) were observed by the appearance of redox peaks at +1.2 V and +0.4 V in the CVs of poly(Th). The peak current of redox peaks increased continuously with increasing potential cycles and implying the continuous build up of poly(Th) on the working electrode (ITO). In order to introduce epoxy group as active site for immobilization of enzyme, we coated the poly(Th-*co*-epoxyTh) on the surface of ITO electrode. In Fig. 1(b), the starting oxidation peak of comonomer with Th and EpoxyTh was appeared at +1.5 V, and the doping/dedoping processes of poly(Th) were observed by the appearance of redox peaks at +1.4 V and +0.45 V in the CVs of poly(Th-*co*-EpoxyTh). The peak current of redox peaks increased continuously with increasing potential cycles and implying the continuous build up of poly(Th-*co*-EpoxyTh) on the working electrode (ITO).

**Figure 2.**
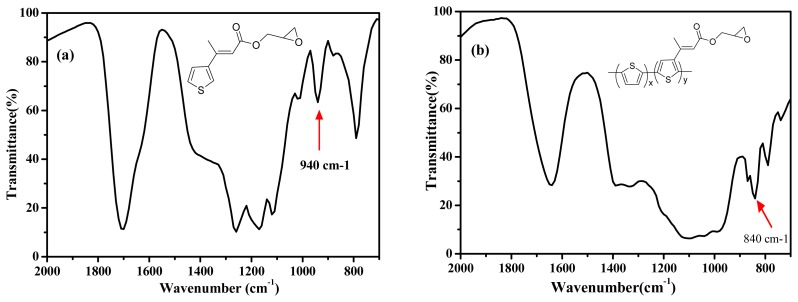
FTIR spectra of EpoxyTh monomer (a), and poly(Th-*co*-EpoxyTh) film (b).

Figure 2 shows the FTIR spectra of the EpoxyTh monomer (a) and poly(Th-*co*-EpoxyTh) film (b). In Figure 2(a) of EpoxyTh, a characteristic peak at 1700 cm^□1^ assigned to carbonyl group of EpoxyTh was observed. The epoxy group of monomer at 940 cm^−1^ and poly(Th-*co*-epoxyTh) on ITO at 840 cm^−1^ was appeared. These results clearly indicate that the active epoxy group for immobilization of enzyme was successfully introduced on polymer electrode.

**Figure 3.**
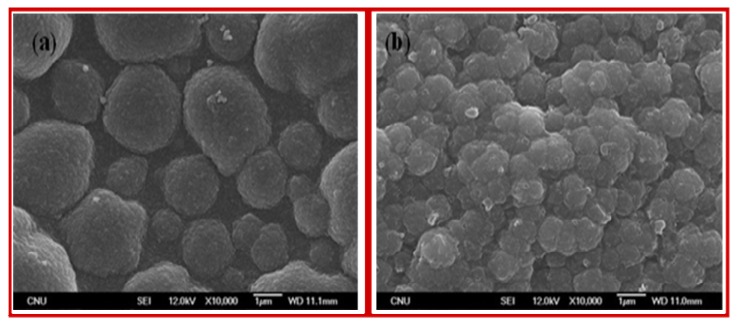
SEM images(* 10,000) of poly(Th) (a), and poly(Th-*co*-EpoxyTh) (b).

**Figure 4.**
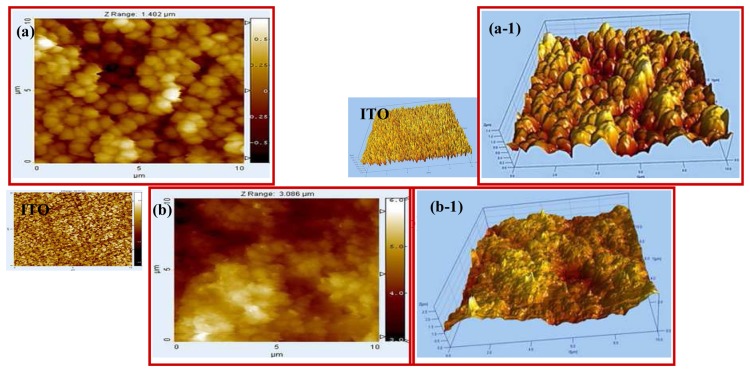
AFM and 3D images(10 mm scale) of poly(Th) (a)/(a−1), and poly (Th-co-EpoxyTh) (b)/(b−1).

Figure 3 presents the SEM images of the polymer electrodes such as poly(Th) (a), and poly(Th-*co*-epoxyTh) (b) on ITO electrode prepared by electrochemical copolymerization. The surface morphology of poly(Th) electrode appeared with typically roughness and fractal-like growth patterns. On the other hand, the surface morphology of poly(Th-*co*-EpoxyTh) electrode was shown as like small fractal growth patterns. AFM images [Figure 4(a, b)] and 3D projection of AFM [Figure 4(a-1,b-1)] corresponding to the surface profile of poly(Th) and poly(Th-*co*-EpoxyTh) were presented, respectively. The surface profile of poly(Th) film was the rough with fractal-like patterns, while the surface morphology of poly(Th-*co*-EpoxyTh) was dramatically changed to smooth fractal behavior. Poly(Th-*co*-EpoxyTh) has large features with sizes larger than observed for the surface of poly(Th).

**Figure 5.**
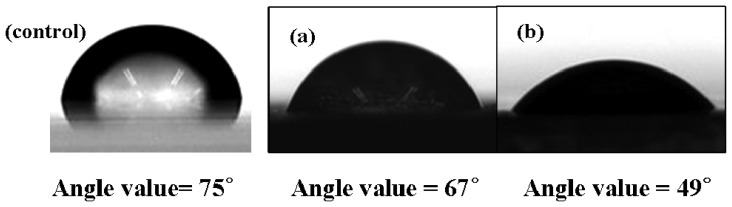
Water contact angle for non-treatment ITO glass (control), poly(Th) (a), and poly(Th-*co*-EpoxyTh) (b).

Figure 5 represents the contact angle measurement images of poly(Th) (a) and poly(Th-*co*-EpoxyTh) electrode (b). The contact angle of water at ITO glass surface was 75°. An increase in contact angle (67°) was noticed with a deposition of poly(Th). At the poly(Th-*co*-EpoxyTh) surface, the contact angle become much lower than (49°) than at poly(Th) surface. Thus, the changes in surface roughness between the polymer surface of poly(Th) and poly(Th-*co*-TAA) as noticed by AFM images (Figure 3 and 4) and the functional groups present the surface of poly(Th-*co*-EpoxyTh) alter the contact angle by modifying the surface energy. Thus, the functionalized polymer electrodes were successfully prepared to tune the surface properties of the electrodes. The surface of poly(Th-*co*-EpoxyTh) was more hydrophilic in comparison to poly(Th) electrode. So, HRP could be loaded more effectively. Practically, the contact angle of HRP-immobilized electrode was at 0°.

**Figure 6.**
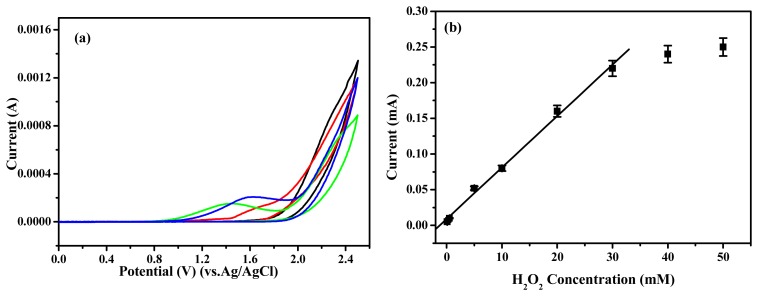
Cyclic voltammogram of H_2_O_2_ sensing from enzyme modified poly(Th-*co*-EpoxyTh) electrode (a) at 5 mM (black), 10 mM (red), 20 mM (blue), and 30 mM (green) of H_2_O_2_, and the calibration plot of the above electrode (b) according H_2_O_2_ concentration.

Many researchers published about the reduction of H_2_O_2_ using immobilized HRP as catalysis [16-18]. So, we obtained the chemical performance of the biosensor with HRP for H_2_O_2_ reduction. The HRP-immobilized polymer electrode was tested for the sensing of H_2_O_2_ in a phosphate buffer (pH=7.0) and calibration plot of the HRP-immobilized polymer electrode was obtained by increasing H_2_O_2_ concentration. As shown in Figure 6, the current increased as increasing the H_2_O_2_ concentration. The HRP-immobilized electrode showed linearity from 0.1 to 30 mM (r^2^=0.995) and the detection limit was calculated to be 0.03 mM based on three times measurement for the standard deviation of the blank noise (95% confidence level, n=3). The detection limit can be comparable to polyaniline based electrode [19]. It is to be noted that a much lower current could be noticed for poly(Th-*co*-EpoxyTh) electrode to the buffer solution without having H_2_O_2_. A significantly high current response was noticed for HRP-immobilized polymer electrode at 1.6 V with a peak maximum of 0.22 mA at 30 mM. It showed that the HRP was saturated. This clearly demonstrates the excellent electrocatalytic performance of the HRP-immobilized polymer electrode towards sensing of H_2_O_2_, and the HRP-immobilized poly(Th-*co*-EpoxyTh) electrode can be used to detect H_2_O_2_. The fabrication reproducibility of nine electrodes, made independently, showed an acceptable reproducibility with a variation coefficient of 3.3% for the current determined at 3 mM H_2_O_2_. By a calibration curve method, the recoveries of four H_2_O_2_ samples in the range of 2∼50 mM was calculated. The average recovery was 97% with a variation coefficient of 3.7%. Good reproducibility may be explained by the fact that the HRP molecules are more firmly attached on the surface of the poly(Th-*co*-EpoxyTh) polymer compared to the Au-polythiophene based electrode [14]. The above H_2_O_2_ sensors were stored in 0.1 M phosphate buffer (pH 7.0) in refrigerator when not in use. The response of these sensors decreased to 90% after storing for 1 month.

## 4. Conclusions

Polymer electrodes such as poly(Th) and poly(Th-*co*-EpoxyTh) electrodes were fabricated by electro-oxidative polymerization and copolymerization. The functional groups in the surface of the polymer films alter the physical and chemical properties of the surfaces and make the surface suitable for loading enzymes. The enzyme modified electrode exhibits augmented electrocatalytic property of the detection of H_2_O_2_. Also, the functional modified electrode provide scope for fabrication of a mediator free biosensor electrode.
